# Agronomic Productivity and Organic Fertilizer Rates on Growth and Yield Performance of Cabbage (*Brassica oleracea* var*. capitata* L.) in Northwestern Ethiopia

**DOI:** 10.1155/2022/2108401

**Published:** 2022-06-09

**Authors:** Yohannes Gelaye, Esubalew Tadele

**Affiliations:** College of Agriculture and Natural Resources, Debre Markos University, P.O. Box 269, Debre Markos, Ethiopia

## Abstract

Cabbage (*Brassica oleracea* var. *capitata* L.) is a popular leafy vegetable in Ethiopia. However, the production and productivity of the crop are often constrained by several factors, such as deprived soil fertility and poor agronomic practices. Thus, a study was conducted in two locations in the East Gojjam zone of northwestern Ethiopia during the 2021/2022 cropping season to evaluate the effect of bud numbers and farmyard manure fertilizer rates on the growth and yield components of cabbage. Three numbers of cabbage buds (1, 2, and 3) and four levels of farmyard manure (0, 2.5, 5, and 7.5 tons/ha) were laid out in a 3 × 4 factorial arrangement in a randomized complete block design with three replications. Data on yield and quality were recorded and subjected to analysis of variance. The results revealed that growth, yield, and quality components were significantly influenced by the interaction effects of bud number and farmyard manure fertilizer rate. In both locations, the highest marketable (41.8 tons/ha) and total (43.1 tons/ha) yields were attained from the combined effects of 2 buds of cabbage and 5 tons of farmyard manure. The highest medium-sized heads (31.8 tons/ha) of cabbage were also recorded from the combination of 2 buds with 5 tons of farmyard manure. Moreover, the combined effects of 2 buds and 5 tons of farmyard manure showed the highest net benefit (5,679.03 US dollars) over the other treatments at the two locations. Hence, based on the results of the study, the combination of 2 buds and 5 tons of farmyard manure fertilizer can be suggested for the economical production of cabbage in northwestern Ethiopia and similar environments.

## 1. Introduction

Cabbage (*Brassica oleracea* var. *capitata* L.) is the fifth most important vegetable crop belonging to the family Cruciferae, and it is a biennial crop with overlying leaves from a compact head [1]. *Brassicaceae* is an important and highly diversified group of crops grown worldwide [2]. The beginning of cabbage was in Western Europe and the North Sea shorelines of the ocean, and it was domesticated and used for human consumption from the earliest antiquity [3].

It is a cool-season crop that is popular with commercial producers [4]. However, it can be cultivated anywhere in the world for use in fresh and processed forms [5]. Additionally, cabbage is a known vegetable crop worldwide because of its adaptability to a wide range of climate and soil conditions. Ethiopia has appropriate edaphic and climatic conditions for the production of cabbages [6]. Cabbage prefers light sand to heavier clay soils with high organic matter content [7]. The ideal soil pH ranges from 5.5 to 6.5 [8]. In soils with a pH above 6.5, the leaves become dark, and the edges of the leaves die [9]. Cabbage demands even moisture to produce good heads, and it requires 380 to 500 mm depending on the climate and length of the growing season.

Due to its anti-oxidant, anti-inflammatory, and anti-bacterial properties, cabbage is broadly utilized in conventional medicine to alleviate signs and symptoms related to gastrointestinal disorders [10]. Nutritionally, 1 cup of uncooked cabbage consists of 93% water and is a great supply of nutritional fiber and nutrients [11].

Cabbage is grown for its head in over ninety countries worldwide. China, India, and South Korea are the major cabbage-growing countries in the world, and Kenya, Egypt, Ethiopia, Niger, and South Africa are the top 5 cabbage producers in Africa [12]. Cabbage is the second most important vegetable crop in Ethiopia, both in area coverage and production, after red pepper [13]. According to the Central Statistical Agency (CSA) 2017/2018 annual report, cabbage production in Ethiopia, the Amhara region, and the eastern Gojjam area are predicted at 38,681.45 tons, 6,276.43 tons, and 1,988.69 tons, which are produced on 6,188.56 ha, 895.98 ha, and 219.65 ha, with mean productivity of 6.25 tons/ha, 7.0 tons/ha, and 9.05 tons/ha, respectively. However, the world average yield is 10.40 tons/ha [14].

Because of the limited availability and high cost of chemical fertilizers, most smallholder farmers in tropical regions use inadequate inorganic fertilizers and poor agronomic practices for crop production [15]. Most smallholder farmers in Ethiopia use below the recommended rates and out of the crop requirement [16]. On the other hand, organic fertilizers and agronomic practices are still a primary source of mineral elements and management options, particularly for the resource-poor farmers of developing countries [17]. However, the use of organic fertilizers such as farmyard manure for crop production depends largely on the usual farming practice. Depending on different conditions, the physical and chemical characteristics of the soil vary from one area to another. Therefore, the choice of the growing environment is probably the most critical decision concerning equivalent cabbage quality with the intended market. Even only a local variety called the Copenhagen market with no application of improved technologies, such as fertilizer and other agronomic practices (like bud management), is produced in several districts of northwestern Ethiopia (personal observation). However, farmers are aware of the importance of the crop and looking for better technologies that improve the performance of the crop. Regarding the number of buds, no research work has been carried out. However, some research results reported that cabbage has several buds on its stem, and after harvesting the first crop, ancillary buds can be initiated and developed into the head and then used for making salads [18]. In the approach of scientific research, this practice is not well explained and is not yet justified. Of course, the cabbage crop has several auxiliary buds on its stem [19]. In either case, these buds are responsible for developing into smaller heads. After normal harvesting of cabbage yield or mechanical damage to the head, new buds are started to develop into smaller heads on the single stem of the cabbage crop. On the other hand, organic fertilizers are an important source of plant nutrients but contain relatively small amounts, which are not readily available [20]. Thus, the combination of farmyard manure rates and bud number is likely to be more productive and economical for cabbage production. In addition, the nutrients contained in farmyard manure are released more slowly and are stored longer in the soil, thus ensuring a long residual effect and promoting better root development and leading to higher yields [21]. Similarly, even if the practice is not yet common, determining the number of buds by pinching practice is very simple and can play an important role in improving cabbage production. The application of farmyard manure fertilizer alone has been reported to be insufficient to produce cabbage. Therefore, there must be proper integration and combination with a better agronomic practice. The integration of this practice can improve the productivity of cabbage and the longevity of the soil. The potential of horticultural crops, especially vegetables, is untapped due to a lack of improved varieties, inadequate agronomic practices, biotic and abiotic stresses, and imbalanced soil nutrients [22]. To date, research institutions in Ethiopia have released many varieties of horticultural crops, including cabbage. However, these technologies have not been fully packaged for farmers. Accordingly, the lack of improved agronomic practices is the key production limitation. Therefore, this study was initiated to evaluate the effect of agronomic productivity and organic fertilizer rates on the growth and yield performance of cabbage. Specifically, the objectives of this study were as follows:

- Determining optimum bud number and farmyard manure () rate for an ideal yield of the cabbage crop
- Evaluating the interaction effects of bud numbers and FYM fertilizer rates on growth, yield, and quality of cabbage

## 2. Materials and Methods

### 2.1. Area Description

The experiment was conducted in two locations in the East Gojjam area, namely, Sinan (Location 1), Gedamawit Kebele on a farmer's field, and Debre Markos (Location 2), Debre Markos University Research and demonstration site, under rain-fed conditions in 2021/2022. Gedamawit Kebele is geographically located between 10°17′00″ to 10°21′30″ north latitude and 37°42′00″ to 37°45′30″ east longitude, and its elevation varies in the range of 2,350–3,358 m above sea level. Debre Markos is also located between 10°17′00″ to 10°21′30″ north latitude and 37°42′00″ to 37°45′30″ east longitude, and the altitude varies from 2,350 to 2,500 meters above sea level.

Both locations have relatively similar annual rainfalls of 1,380 mm and 15°C and 22°C minimum and maximum temperatures, respectively. Additionally, in both locations, the rainy season lasts from mid-May to mid-September, with maximum rainfall in July and August (Table 1).

Although there are a variety of soil types in both locations, the most dominant one is nitisol [23] (Table 2).

### 2.2. Experimental Materials, Treatments, and Design

#### 2.2.1. Experimental Materials

Copenhagen market varieties, well adapted to a wide range of climatic conditions and altitudes, were used as planting materials. Farmyard manure was used for fertilization. For all of the treatments, an equal amount of urea fertilizer (100 kg/ha (46% N)) was used and applied in the ring application method.

#### 2.2.2. Experimental Treatment and Design

The treatment consisted of three bud numbers, namely, without pinching (normal), pinching with two and three buds, and four farmyard manure levels (0, 2.5, 5, and 7.5 tons/ha). The experiment was designed in a 3 × 4 factorial design using a three-replication randomized complete block design, and each plot had a net plot area of 2.16 m^2^. The spacing between rows and plants was 0.4 m and 0.3 m, and the distances between plots and adjacent blocks were 0.5 m and 1 m, respectively. A total of 12 treatments were randomly assigned to the experimental plots [24]. The total number of experimental plots was 36 (12 treatments × 3 replications).

### 2.3. Experimental Procedures and Management Activities

The nursery bed was made from finely prepared soil mixed with well-decomposed farmyard manure [1]. The size of the bed was 1.0 × 3.0 m. Following the recommendation, cabbage seeds were sown in the nursery site at Debre Markos, and seed sowing was performed inline [24]. In parallel, old animal manure was collected and composted in a pit for a month to produce farmyard manure. It was applied to the field and mixed during land preparation ahead of a month (first week of June 2021) [25]. When the seedlings attained a height of 15 cm with 3–4 leaves, transplanting was performed (on the fourth week of June 2021). During transplanting, pinching was performed on a cut or pinch section (2-3 cm) equally by letting the seedlings for the control treatment. The spacing between rows and plants was 0.4 m and 0.3 m, respectively. The spacing between plots and adjacent blocks was 0.5 m and 1 m, respectively. The seedlings were planted on the raised bed to provide good drainage. Each plot had an area of 2 m × 2.4 m = 4.8 m^2^. A total of 40 plants/plot were used for planting. After 15 days of transplanting (successful establishment), bud numbers were determined by thinning (agronomic practices) by letting 2 and 3 buds per plant. After that, all management practices (hoeing and weeding) were performed equally as per the recommendations [25]. Cabbage aphids were controlled by using dimethoate 40% EC chemical, and spraying was performed at 2-week intervals. Urea was applied equally in two splits using the recommended rate of 100 kg/ha [26].

### 2.4. Data Collection

The following yield and yield-related parameter data were collected, and the collection was done from the middle row, leaving aside plants in the border row to avoid border effects.

#### 2.4.1. Phenological Parameters

Days to 50% head initiation (HI; number): It was recorded when half of the plants in a net plot were heads.

Days to 90% maturity of heads (HM; number): It was recorded from the date of transplantation until 90% of the heads of the net plot reached maturity.

#### 2.4.2. Growth Parameters

Plant height (PH; cm): It was measured by placing a ruler from the ground to the top of the longest outer head of an individual plant at 90% days to head maturity. Therefore, the mean of six selected plants from a single plot was recorded and expressed in centimeters (cm). The mean values were used for further analysis.

#### 2.4.3. Yield Parameters

Head weight (HW; g): It was measured by weighing the heads per plant with a sensitive balance. Head fresh weight per plant was recorded from sample plants and expressed in grams. The mean values were used for further analysis.

Marketable yield (MY; tons/ha): It was measured by weighing insect- and disease-free, mechanically undamaged heads weighing >0.5 kg from the area of the net plot with a sensitive balance. The mean values were used for analysis and then converted to tons per hectare.

Unmarketable yield (UY; tons/ha): It was measured by weighing diseased, mechanically damaged, or injured heads and weighing <0.5 kg using a sensitive balance and then converted to tons per hectare.

Total yield (TY; tons/ha): It was recorded as the sum of marketable and nonmarketable head yields, and the result was converted to tons per hectare.

#### 2.4.4. Quality Parameters

Compactness index (CTM; %): It was measured according to standard procedures.

Head size distribution by weight (tons/ha): Cabbage was arranged by weight. With this in mind, heads in the weight category of >1.0 kg, 1–0.5 kg, and <0.5 kg weight classes were considered large (L), medium (M), and small (S), respectively [27]. Consequently, the heads harvested from the net area of the plot were proportionally weighted with a sensitive balance and expressed in tons per hectare.

### 2.5. Data Analysis

The experimental data were analyzed by using analysis of variance using R software version 4.1.2 (2021-11-01). During the analysis process, the first separate analysis was performed for each location. The homogeneity of error variances for the error means square values of all parameters were tested using the variance ratio test method. Since the variance ratio of all crop components was lower than three, a combined analysis method was used [28]. The interpretations were made following the statistical procedures for agricultural research [29]. The least significant difference (LSD) was used to separate means whenever the analysis of variance showed a significant difference between the treatment means.

### 2.6. Cost-Benefit Analysis

The cost-benefit analysis was done to estimate the relative economic returns of the applied treatments using the prevailing market prices. The marketable yield of cabbage was adjusted by a 10% downscale value to manage the variability between a researcher and a farmer [30]. The cost of farm services was taken from the Debre Markos and Rebu Gebeya towns in northwestern Ethiopia. The average value was used for the calculations and further analysis. The economic indicators used were as follows:

#### 2.6.1. Gross Benefit

This is the product of the adjusted yield (tons/ha) and sale prices. It was calculated by multiplying the yield in tons/ha by the market price.

#### 2.6.2. Net Benefit

It was estimated by subtracting the total cost of production from the gross benefit.

#### 2.6.3. Marginal Analysis

This compares the net benefits with the total variable costs. The total variable cost was determined for each treatment and compared with the net benefit.

#### 2.6.4. Dominance Analysis

Treatments were arranged in terms of variable costs from the lowest to the highest costs (cost increasing order). The equivalent net benefits were also indicated. A treatment is dominant when it has a higher cost but a lower net benefit than any of the preceding treatments.

#### 2.6.5. Marginal Rate of Returns

It is the percentage change in benefit over the change in total variable cost in moving from a lower-cost treatment to a higher one. All treatments were arranged from the highest to the lowest in terms of profitability. This has been achieved by dividing the total variable cost by the net benefit multiplied by 100.

## 3. Results

**Figure 1:**
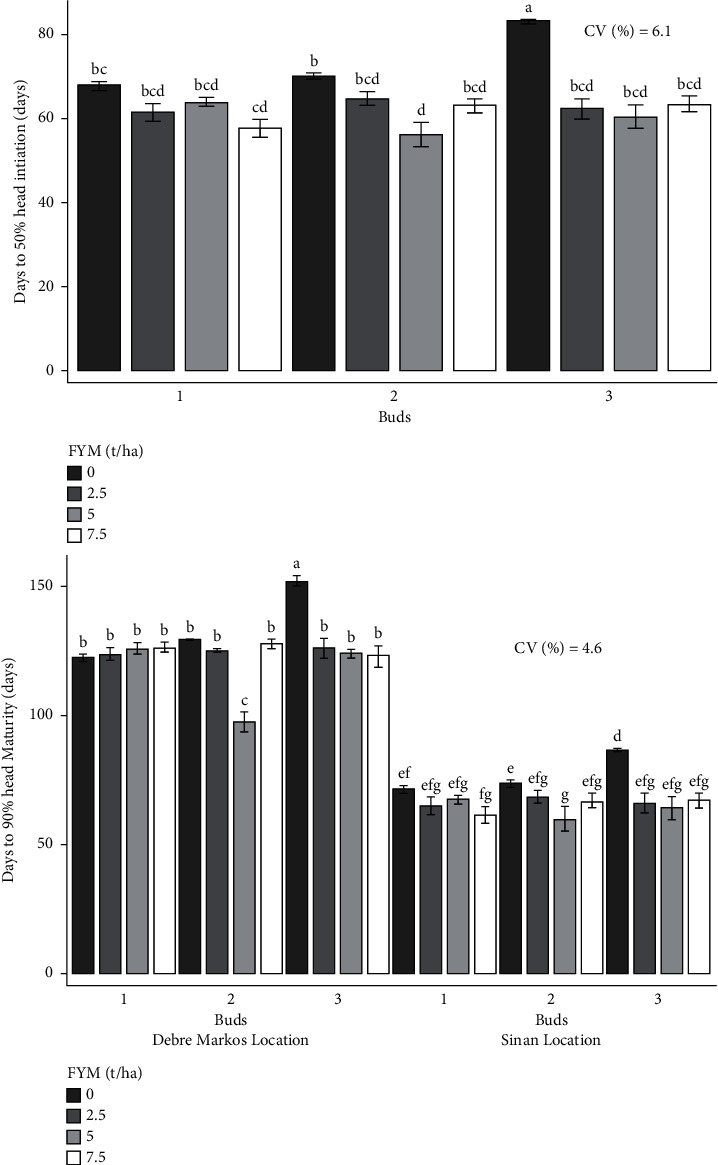
Interaction effects of bud numbers and farmyard manure on cabbage phenology.

**Figure 2:**
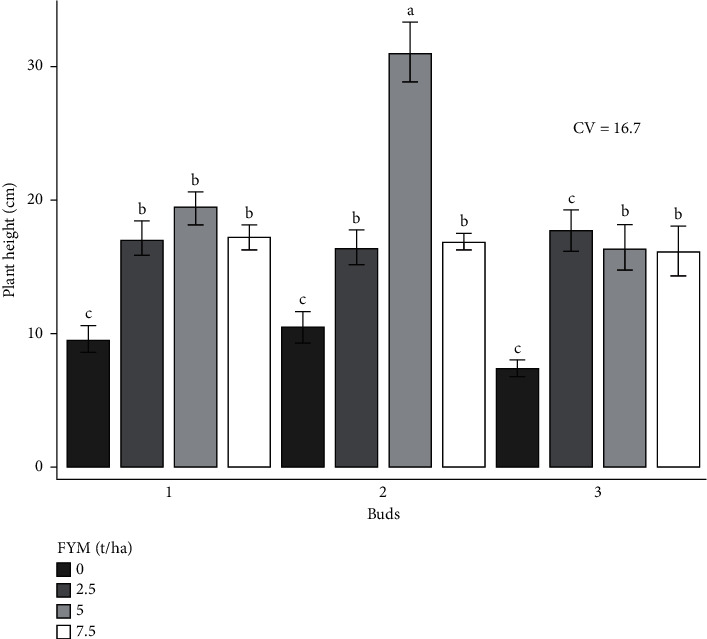
Plant height as influenced by the interaction effect of bud numbers and farmyard manure fertilizer rates.

**Figure 3:**
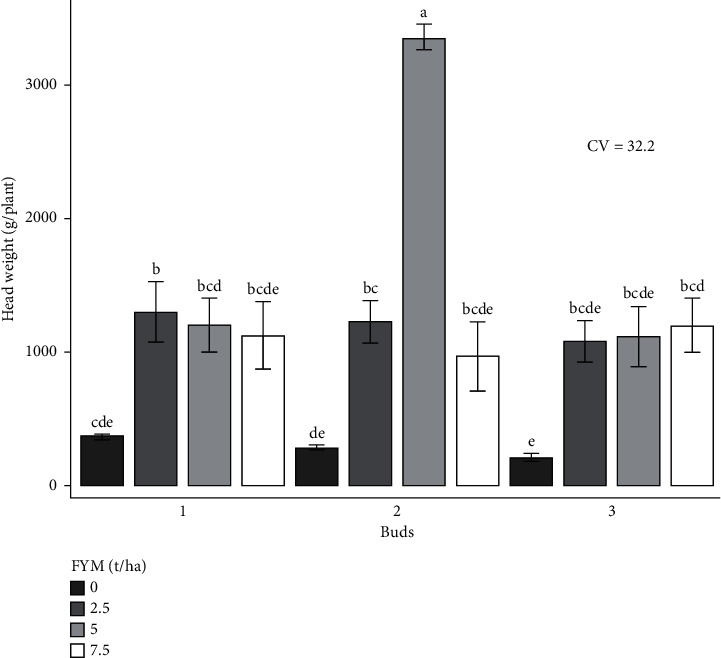
Head weight as influenced by the interaction effect of bud numbers and farmyard manure fertilizer rates.

**Figure 4:**
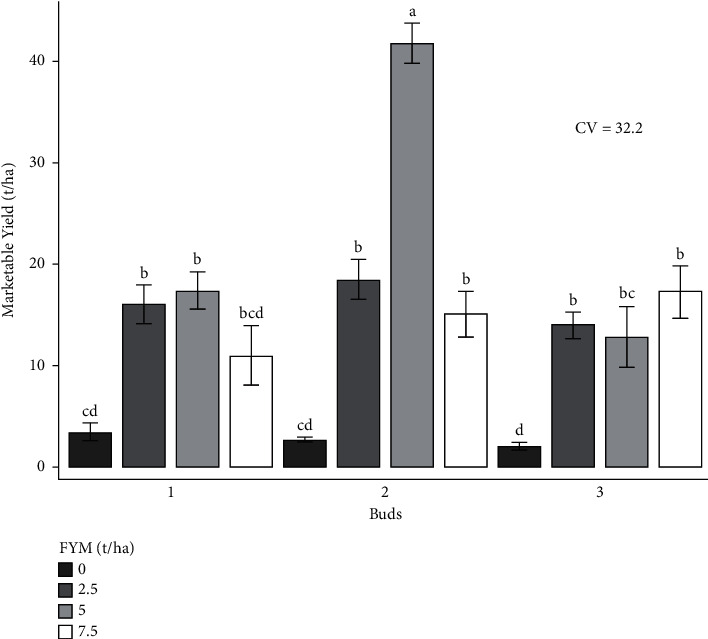
Marketable yield as influenced by the interaction effect of bud numbers and farmyard manure fertilizer rates.

**Figure 5:**
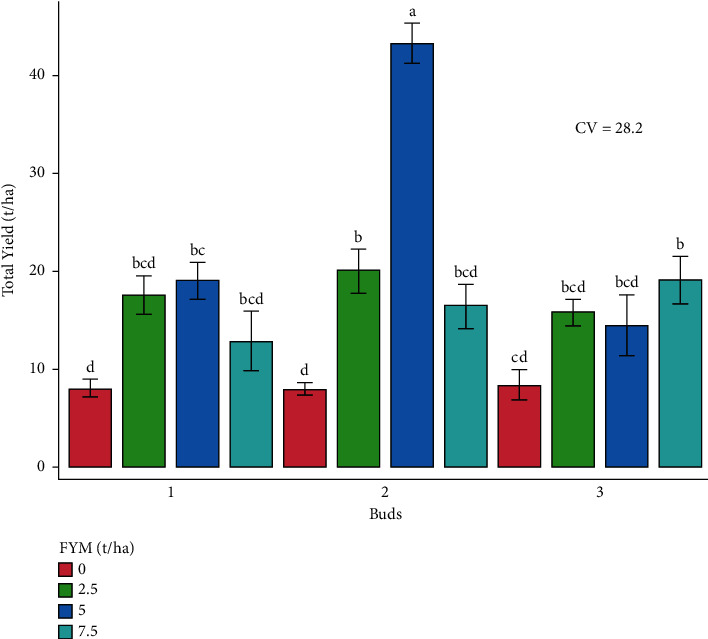
Total yield as influenced by the interaction effect of bud numbers and farmyard manure fertilizer rates.

**Figure 6:**
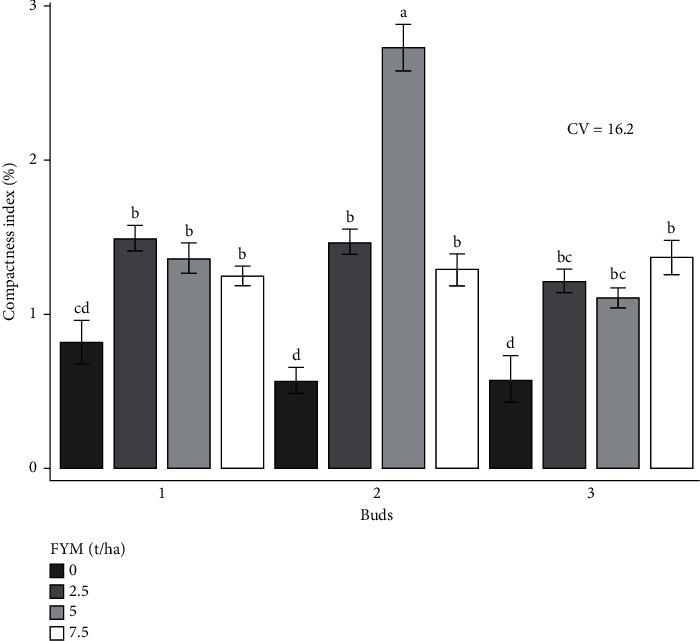
Compactness index as influenced by the interaction effect of bud numbers and farmyard manure fertilizer rates.

The experiment was performed to determine the combined effects of bud number and farmyard manure on cabbage growth and yield. As a result, the effects of bud number and farmyard manure, as well as their interactions, on cabbage growth and yield are presented and discussed in different figures and tables (Figures 1–6 and Table 3).

### 3.1. The Effect of Bud Number and FYM Rate on Cabbage Crop Phenology

#### 3.1.1. Days to 50% Head Initiation and 90% Maturity

The analysis of variance showed that head initiation and maturity of cabbage were significantly (*p* < 0.01) influenced by the interaction effects of bud number and rate of farmyard manure fertilizer (Table 4). At both locations, the combined application of 2 buds and 5 tons of farmyard manure resulted in the shortest day of head initiation (56 days) and maturity (78.5 days). The longest head initiation (83 days) and maturity (119.3 days) were recorded from the combined effects of 3 buds with no fertilization of farmyard manure (Figure 1).

### 3.2. The Effect of Bud Number and Farmyard Manure Rate on Cabbage Crop Growth

#### 3.2.1. Plant Height (cm)

Bud number and farmyard manure fertilizer rate influenced the height of the cabbage very highly significantly (*p* < 0.001; Table 4).

The tallest plant (30.9 cm) was attained from the combined treatment of 2 buds and 5 tons of farmyard manure fertilizer. The shortest plant (7.4 cm) was recorded from 3 buds with the control treatment of farmyard manure, which was also statistically similar to the results of other treatments (Figure 2).

### 3.3. The Effect of Bud Number and Farmyard Manure Rates on Cabbage Crop Yield

#### 3.3.1. Head Weight (g)

A significant difference (*p* < 0.01) in the head weight (g) of the cabbage crop was observed for the interaction effects of bud number and farmyard manure fertilizer rate (Table 4). Thus, the combined application of bud number and farmyard manure fertilizer rate resulted in the highest head weight (3,355 g), whereas the lowest (217.3 g) was recorded from 3 buds with no farmyard manure application in both locations (Figure 3).

#### 3.3.2. Marketable Yield (tons/ha)

The combination of bud and farmyard manure fertilizer rates revealed a very high significant difference (*p* < 0.001) in marketable yield in both locations (Table 4).

Compared to other treatments, the highest marketable yield (41.8 tons/ha) was attained from the two locations of 2 buds and 5 tons of farmyard manure fertilizer, and the lowest (2.1 tons/ha) was recorded from 3 buds with no farmyard manure application in both locations (Figure 4).

#### 3.3.3. Unmarketable Yield (tons/ha)

The bud number and farmyard manure interaction effect also showed a significant difference (*p* < 0.01) in the unmarketable yield of cabbage (Table 4).

In this case, the highest volume of unmarketable yield (6.3 tons/ha) was recorded from the 3 buds of cabbage with no farmyard manure application technique. However, the smallest unit (1.3 tons/ha) of yield was recorded from 2 buds of cabbage to 7.5 tons of farmyard manure in both locations (Table 3).

#### 3.3.4. Total Yield

The influence of the bud and farmyard manure rates on the total yield revealed a very high significant difference (*p* < 0.001) (Table 4). The highest yield (43.1 tons/ha) was harvested in both districts, with the treatment combination of 2 bud numbers and 5 tons of farmyard manure producing the highest total yield per hectare. The lowest total yield (7.9 tons/ha) was harvested from 2 buds and no application of farmyard manure fertilizer, and it was statistically similar to the yields harvested from 3 buds and no farmyard manure application (Figure 5).

### 3.4. The Effect of Bud and Farmyard Manure Rates on Cabbage Crop Quality

#### 3.4.1. Compactness Index

It was significantly (*p* < 0.01) influenced via the interaction effect of cabbage bud number and the rate of farmyard manure (Table 4). The highest compactness index (2.7%) was attained with 2 buds and 5 tons of farmyard manure fertilizer rates in both locations. The lowest index (0.5%) was recorded from a combination of 2 and 3 buds with no fertilization (Figure 6).

#### 3.4.2. Cabbage Head Size Distribution by Weight

*(1) Small-Sized Heads*. Bud number and farmyard manure fertilizer applications revealed a significant difference (*p* < 0.01) in small cabbage heads (Table 4). The smallest volume of small-sized heads (1.3 tons/ha) was recorded from 2 buds with 7.5 tons of farmyard manure. On the other hand, the highest (6.3 tons/ha) quantities of small-sized heads were recorded from 2 and 3 buds of cabbage with no fertilization activity in both locations (Table 3).

**Figure 7:**
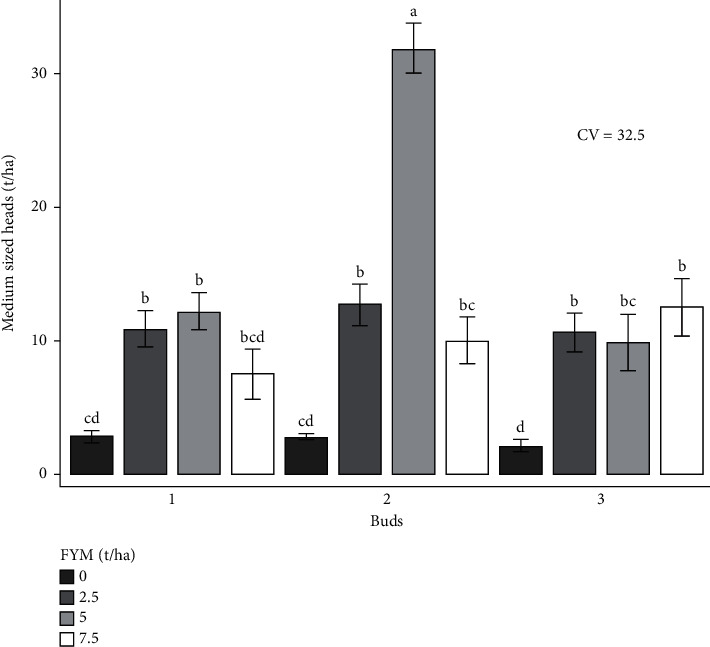
Medium-sized heads as influenced by the interaction effect of bud numbers and farmyard manure fertilizer rates.

*(2) Medium-Sized Heads*. A very high significant difference (*p* < 0.001) was observed in the medium-sized heads for bud number and farmyard manure fertilizer interaction (Table 4). High medium-sized heads (31.8 tons/ha) were attained from the combined effects of 2 buds and 5 tons of farmyard manure fertilizer rates in both locations. The lowest (2.1 tons/ha) was recorded from 3 buds of cabbage with zero level of fertilizer (Figure 7).

**Figure 8:**
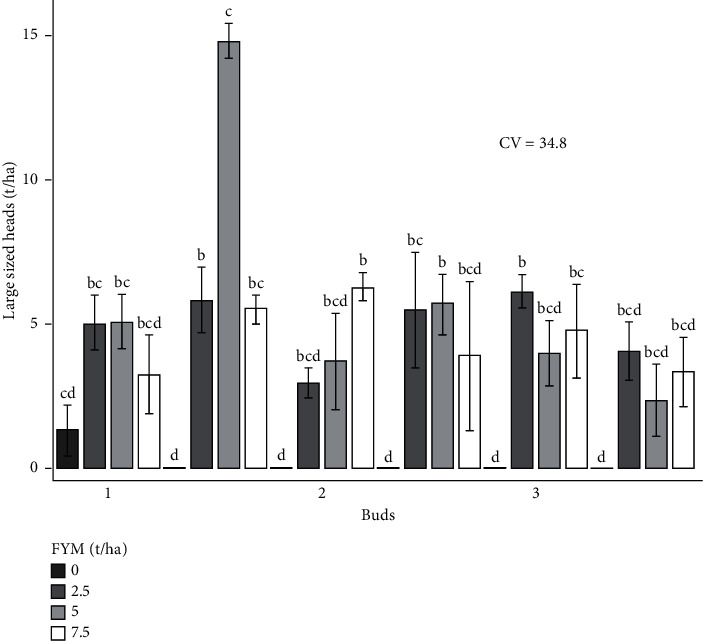
Large-sized heads as influenced by the interaction effect of bud numbers and farmyard manure fertilizer rates.

*(3) Large Heads*. The interaction effect of buds and farmyard manure fertilizer rates on large heads revealed a significant difference (*p* < 0.01) at both sites (Table 4). The highest quantity (14.8 tons/ha) was attained from the combination of 2 buds and 5 ton hectares. However, insignificant amounts were recorded from 2 and 3 buds with no fertilization processes (Figure 8).

## 4. Discussion

### 4.1. The Effect of Bud Number and Farmyard Manure Rate on Cabbage Crop Phenology

#### 4.1.1. Days to 50% Head Initiation and 90% Maturity

The findings revealed that high treatment resulted in a delay in the onset of various reproductive growth phases, whereas low-nutrient or no-nutrient treatments resulted in a dramatic acceleration in the onset of various reproductive growth phases. This result was in agreement with the report that a significantly shorter time (57.45 days) for cabbage head initiation was recorded from a treatment that received nitrogen and FYM fertilizer ahead of the control treatment [31].

According to the analysis results, the days to head initiation were inversely related to the nutrient rates. As the combined rate increased, the number of days with 50% head formation decreased. In another explanation, the earlier the crop head forms, the more nutrients we use. Greater fertility levels preferred head cabbage onset and maturity [32]. On the other hand, plants with no fertilizer application required a longer time (77.0 days) to initiate their heads [33]. Similarly, there was a shorter head initiation time in cabbage plants that received organic and inorganic fertilizers [34].

Usually, cabbages that received the highest farmyard manure rates in buds of cabbage extended maturity earlier than those receiving no or low farmyard manure rates [35]. Additionally, the result is in agreement with the report that the combined use of organic and inorganic nutrients was associated with delays in plant maturity [36].

Contrary to farmyard manure, it accelerates plant maturity, and thereby, the cabbage plants treated with the highest nutrient availability matured physiologically earlier than the untreated treatments [37]. Additionally, the application of organic manure influenced the longevity of vegetables due to the increased nutrient uptake by the plants and greater development of water-conducting tissues [38]. The effect of fertilization on the maturity of cabbage heads where fertilizer application reduced the date of maturity compared to without fertilization.

### 4.2. The Effect of Bud Number and Farmyard Manure Rate on Cabbage Crop Growth

#### 4.2.1. Plant Height (cm)

In general, the type of organic fertilizer did not affect the growth of the cabbage crop (number of leaves and plant height) as much as it influenced the yield components (fresh and dry mass) and quality components (grading and head diameter). As expected, the rate of organic fertilizer application significantly influenced almost all parameters measured. Increasing plant height in farmyard manure treated with two buds of the plot could be expedited by improving soil conditions such as nutrient content, water retention capacity, and composition [39]. Farmyard manure may stimulate plant growth, which might be supported by the fertilizer's auxins, cytokinins, and gibberellins [40]. Using only inorganic fertilizer is detrimental to soil health. As a result, substituting organic fertilizers for chemical fertilizers and applying farmyard manure may yield better results in terms of increasing cabbage height [41]. Organic fertilizers can be used in place of mineral fertilizers to improve soil structure and microbial biomass. Farmyard manure fertilizer stimulates soil microbial activity, resulting in more nutrient addition for bud growth and, inevitably, more yield [42]. According to the data, the plant height has an increasing trend in growth when the rate of farmyard manure with two buds is increased. In agreement with the results, the height of cabbage was observed to be high as the rate of farmyard manure increased [43]. Additionally, the increased use of nitrogen and farmyard manure leads to increased plant height [44].

In general, the increase in plant height caused by farmyard manure application could be attributed to the fact that organic manure improves soil structure and aggregation, which can improve nutrient supply [45].

### 4.3. The Effect of Bud Number and Farmyard Manure Rates on Cabbage Crop Yield

#### 4.3.1. Head Weight

Relative to the remaining treatments, the application of the highest dose of farmyard manure with two buds attained a relatively high head weight, which was far higher than that of the control and overfertilized treatments. Weight increased significantly with manure application to the optimum bud combination of cabbage. Farmyard manure fertilizer rates with different bud numbers responded differently to the head weight of cabbage [46]. Head weight is one of the most important characteristics for measuring yield performance. Consistent with this finding, increasing farmyard manure with a certain number of buds increased the head weight of cabbage crops [47]. Additionally, organic matter sources significantly increased growth parameters, which in turn synthesized more plant metabolites and increased yield [48].

The rate of farmyard manure increases to a certain level, as does the mass of the head. Furthermore, organic manure activates a wide range of living organisms, which release phytohormones and may stimulate plant growth and nutrient absorption [49]. Therefore, nitrogen is required for the multiplication of these organisms, which is why the use of farmyard manure following inorganic fertilizer improved both growth and yield [50].

#### 4.3.2. Marketable Yield

Marketable yield is the most important issue that persuades farmers to cultivate a cabbage crop. Increases in cabbage yield in response to augmented organic fertilizer levels could be attributed to the positive role of balanced nutrients within the physiology of the plant in cell elongation and enhancing higher than ground vegetative growth, photosynthesis, and partitioning of photosynthates to head development. Thus, the combined use of farmyard manure and 2 buds considerably influenced the character of marketable yield in all probabilities that provide nitrogen, phosphorous, and micronutrients to the crop. Moreover, with two numbers of buds, competition for space, moisture, nutrients, and light was also lower over the 3 buds. All treatment combinations showed an enormous yield increment over the control. Consistent with the analysis result, the addition of farmyard manure at an extreme level with high bud numbers failed to boost substantial yields. The yield was reduced because of the overhead application of farmyard manure fertilizer of five tons for all bud number treatments. Previous studies reported that many vegetables showed a significant yield improvement due to the application of organic fertilizers [51]. Additionally, farmyard manure has a high organic carbon content and macro and small nutrients, such as N, P, K, Ca, Mg, and Fe, which are permanently essential for crop growth [52].

#### 4.3.3. Unmarketable Yield

In general, the lowest marketable yield might be recorded from any of the control treatments having no nutrients applied [53]. Even the minimum yield per hectare could be attained from any of the control treatments in cabbage production [54].

#### 4.3.4. Total Yield

The application of farmyard manure significantly increased the total yield, compactness index, and other components compared with the control treatment. The addition of farmyard manure to the soil may improve the physical, chemical, and biological properties of the soil [55]. The increase in soil organic carbon content can increase nutrient accessibility within the soil. Although bud number determination in cabbage may be a new practice, the farmyard manure rate is a crucial topic that many researchers are engaging in. Therefore, it has a major role in soil management because it is one of the foremost challenges for agricultural systems in the tropics [56]. Generally, the utilization of organic manure not only reduces the need for chemical fertilizers but also provides the necessary supplements and essential nutrients to the plants in addition to increasing the soil properties [57].

### 4.4. The Effect of Bud and Farmyard Manure Rates on Cabbage Crop Quality

#### 4.4.1. Compactness Index

In general, head compactness is a desirable attribute in the sense that more produce can be accommodated in lesser shapes/volumes.

The farmyard manure rates led to significant increases in the morphological characteristics of the cabbage plant (length and stem diameter, head diameter, compactness, and head roundness coefficient). This result is similar to researchers who reported significant increases in plant growth and quality components and stems with the addition of farmyard manure fertilizer rates, as these components are familiar with the yield [58]. Significant increments in the diameter and compactness of the heads were found when fertilized with farmyard manure fertilizer [59]. Additionally, cabbage fertilized with farmyard manure fertilizer revealed a significant change in height, head length and diameter, and compactness index [9].

#### 4.4.2. Cabbage Head Size Distribution by Weight

*(1) Small-Sized Heads*. Smaller cabbage heads are favored by the fresh market and can be gained through the selection of a cultivar suitable for such products as well as cultural practices with a reasonable rate of fertilization [60].

*(2) Medium-Sized Heads*. Medium-sized heads contribute directly to marketable yield and are of economic concern to farmers. Improvement in the quality attributes of head cabbage by the application of organic manure together with better agronomic practice helps in the exudation of growth-promoting substances, which leads to better root development and translocation of carbohydrates to storage organs [61]. Organic fertilizer types did not play an important role; the application rate positively influenced the quality. Hence, an increase in organic fertilizer rates also had a positive effect on cabbage head size and diameter irrespective of the type of organic fertilizer.

*(3) Large Heads*. Fertilization has a significant impact on the morphological characteristics of cabbage [36]. Regardless of the plant population, the application of farmyard manure favors most of the head sizes of the cabbage crop. The number of buds together with the farmyard manure fertilizer rate has considerable sustenance on the portion of head class from small to large (0.5–1.5 kg) [27]. High-density cabbage cultivation results in a more uniform head size, which is an important and desired factor for a single plant harvest, and lighter green color outer leaves, which are preferred by consumers [59]. The impact of plant spacing and fertilizer composition on different vegetable crops has also been reported by several researchers. There are also additional reports stated by many scholars that the use of a large plant population is associated with a reduction in cabbage head size [27].

### 4.5. Cost-Benefit Analysis of Cabbage Production as Influenced by Bud Number and Farmyard Manure Fertilizer Rate

For the economic analysis, the average products of each treatment were taken in favor of the total replications [30]. On the rationale of the marginal rate return value (Table 5), there was a net benefit of 5,679.03 US dollars from the combined application of 2 cabbage buds and 5 tons of farmyard manure fertilizer followed by 2 buds and 2.5 tons/ha, with a net benefit of 2,519.61 US dollars (Table 6). The lowest net benefit of 292.30 US dollars was recorded from 3 numbers of buds with no application of farmyard manure fertilizer (Table 7). The general costs, as well as the net benefit, were increased to a certain level for all treatments.

## 5. Conclusion

The results of the present study generally showed that the integrated and combined application of pinching and thinning practices with farmyard manure fertilizer rates influenced all growth and yield components encompassing marketable and total head cabbage yield. After the pinching and thinning processes of cabbage, the highest marketable yield (41.8 tons/ha) was recorded from the 2 buds of cabbage integrated with 5 tons of farmyard manure in both locations. This investigation fundamentally identified that there is a problem of poor agronomic practice in cabbage production systems, including pinching, which has been overlooked for years due to the perception that cabbage cannot be pinched and that buds cannot be initiated. However, pinching is a common agronomic practice for most horticultural crops, including vegetables and ornamental plants. In general, the highest yield of cabbage (43.1 tons/ha) in both locations was attained from the combined effects of 2 buds and 5 tons of farmyard manure. In addition, this combination also significantly influenced head-size distribution (medium-head-size), and 31.8 tons were achieved in both locations. In addition, the combined effects of 2 buds and 5 tons of farmyard manure revealed the highest net benefit (5,679.03 US dollars) over the other treatments at the two locations. Hence, based on the results of the study, the combination of 2 buds and 5 tons of farmyard manure fertilizer can be recommended for the ideal and economical production of cabbage in northwestern Ethiopia in particular and similar environments in general.

## Figures and Tables

**Table 1:** Mean air temperature, monthly rainfall, soil temperature, relative humidity, and soil moisture in the Sinan and Debre Markos areas.

Experimental sites	Cropping season months	Mean monthly rainfall (mm)	Mean air temperature (°C)		Soil temperature (°C)	Relative humidity (%)	Soil moisture (%)
			Minimum	Maximum			
Lo_1_	June	165	14.30	15.5	18.10	89.02	28.12
	July	397	14.35	15.29	17.32	86.14	25.34
	August	143	14.29	15.45	17.97	87.02	27.95
	September	145	14.77	16.05	18.40	80.04	51.58
	October	45	14.53	15.75	18.19	75.29	36.38

Lo_2_	June		14.27	15.4		91.4	
	July		14.30	15.27		88.7	
	August		14.23	15.01		87.3	
	September		14.09	16.02		80.1	
	October		14.31	15.70		77.8	

Source: Debre Markos University Choke Watershed project office, 2021.

**Table 2:** Soil characteristics of experimental sites.

Locations	Parameters	Amount present	Classifications
Lo_1_	Soil texture	14:64:22 (sand, clay, silt %)	Clay
	pH	5.2	Acidic
	CEC (cation exchange capacity)	20.61 cmol/kg	Medium

Lo_2_	Soil texture	12:56:20	Clay
	pH	5.5	Acidic
	CEC	23.54 cmol/kg	Medium

Source: Debre Markos University Choke Watershed project office, 2021.

**Table 3:** Some yield and quality components of head cabbage as influenced by the interaction effects of bud number and farmyard manure fertilizer.

Location	Bud	FYM	S	UY
Lo_1_ + Lo_2_	1	0	4.5^ab^	4.5^ab^
		2.5	1.5^bc^	1.5^bc^
		5	1.4^bc^	1.6^bc^
		7.5	1.7^bc^	1.7^bc^
	2	0	5.1^a^	5.1^a^
		2.5	1.4^bc^	1.4^bc^
		5	1.5^bc^	1.3^bc^
		7.5	1.3^bc^	1.3^bc^
	3	0	6.3^a^	6.3^a^
		2.5	1.6^bc^	1.6^bc^
		5	1.7^bc^	1.7^bc^
		7.5	1.7^bc^	1.7^bc^

LSD (1%)			2.5	2.4

CV (%)			39.4	38.1

LSD = least significant difference and CV (%) = coefficient of variation in percent.

**Table 4:** Mean squares values for the combined analysis of growth, yield, and quality components of head cabbage.

Factors	df	Parameters										
		PH	Hw	CTM	MY	UY	TY	HI	HM	S	M	L
Lo	1	253.5^*∗∗∗*^	937,308.7ns	1.41ns	118.86ns	0.60ns	102.60ns	74.42ns	58,527.61ns	0.68ns	32.58ns	24.50ns
Bud	2	114.1^*∗∗∗*^	2,118,526^*∗∗∗*^	1.23	485.89^*∗∗∗*^	2.11ns	448.02^*∗∗∗*^	147.34^*∗*^	371.46ns	2.17ns	267.70^*∗∗∗*^	31.92^*∗*^
FYM	3	521.2^*∗∗∗*^	7,728,043ns	3.65	1,383.67^*∗∗∗*^	64.05ns	921.85^*∗∗∗*^	690.46^*∗*^	820.29ns	64.28^*∗*^	714.52^*∗∗∗*^	115.21^*∗*^
Lo ^*∗*^bud	2	3.8ns	5,013.847ns	0.01^*∗∗*^	1.04ns	0.06ns	0.89ns	1.65ns	90.54^*∗*^	0.03ns	18.06	13.28ns
Lo ^*∗*^FYM	3	5.4ns	39,590.24ns	0.01^*∗∗*^	3.82ns	0.66ns	7.07ns	1.66ns	60.81^*∗*^	0.61ns	11.33ns	16.33ns
B ^*∗*^FYM	6	86.7^*∗∗∗*^	2,568,926ns	1.21^*∗∗∗*^	354.25^*∗∗∗*^	1.18^*∗*^	352.66^*∗∗∗*^	144.32^*∗∗∗*^	358.05ns	1.12^*∗*^	219.31^*∗∗∗*^	14.76^*∗∗∗*^
Lo ^*∗*^Bud ^*∗*^FYM	6	2.8ns	3,228.514ns	0.03ns	3.99ns	0.62ns	6.23ns	2.52ns	86.05^*∗∗*^	0.69ns	8.27ns	16.19^ns^
Residuals	48	7.8	208,471.5	0.05	25.66	1.26	27.74	25.46	22.87	1.31	14.53	3.70

df = degree of freedom, Lo = Location, PH = plant height, Hw = head weight, CTM = Compactness index, MY = marketable yield, UY = marketable yield, TY = total yield, HI = head initiation, HM = head maturity, S = small-sized heads, M = medium-sized heads, L = large sized heads, and ns = no significant difference. ^*∗*^, ^*∗∗*^, and ^*∗∗∗*^ = indicated significant, highly significant, and very highly difference, respectively.

**Table 5:** Marginal rate of return (MRR) of cabbage yield as affected by bud number and farmyard manure fertilizer rates in northwestern Ethiopia.

Treatment combinations	TVC (USD/ha)	MC (USD/ha)	NB (USD/ha)	MB (USD/ha)	MRR (%)
1:0	0.00		484.61		
1:2.5	52.88	52.88	2,176.34	1,691.73	61.5
2:2.5	55.76	2.88	2,519.61	343.26	228.8
2:5	108.65	52.88	5,679.03	3,159.42	114.8

MC = marginal cost, MB = marginal benefit, and MRR = marginal rate of return.

**Table 6:** Dominance analysis for cabbage yield as affected by bud number and farmyard manure fertilizer rates in northwestern Ethiopia.

Bud + FYM	TVC (USD/ha)	NB (USD/ha)	B:C ratio
1:0	0.00	484.61	–
2:0	2.88	384.80D	133.6
3:0	3.84	286.92D	74.7
1:2.5	52.88	2,176.34	41.15
2:2.5	55.76	2,519.61	45.17
3:2.5	56.73	1,895.57D	33.41
1:5	105.76	2,289.61D	21.64
2:5	108.65	5,679.03	52.26
3:5	109.61	1,662.69D	15.16
1:7.5	158.65	1,364.42D	8.6
2:7.5	161.53	1,929.23D	11.94
3:7.5	162.5	2,232.88D	13.74

B:C = Benefit-cost ratio.

**Table 7:** Economic analysis of cabbage as influenced by bud number and farmyard manure fertilizer rate.

Lo	Bud + FYM	IC per ha FYM (USD)	AC (USD)	LC for mgt (USD)	TVC (USD)	MY (tons/ha)	ADY (tons/ha)	GI	NB	Rank
Lo_1_ and Lo_2_	1:0	0.00	0.00	0.00	0.00	3.5	3.15	484.61	484.61	10
	1:2.5	48.07	4.80	0.00	52.88	16.1	14.49	2,229.23	2,176.34	5
	1:5	96.14	9.6	0.00	105.76	17.3	15.57	2,395.38	2,289.61	3
	1:7.5	144.23	14.42	0.00	1,158.65	11.0	9.9	1,523.07	1,364.42	9
	2:0	0.00	0.00	2.88	2.88	2.8	2.52	387.69	384.80	11
	2:2.5	48.07	4.80	2.88	55.76	18.6	16.74	2,575.38	2,519.61	2
	2:5	96.14	9.6	2.88	108.65	41.8	37.62	5,787.69	5,679.03	1
	2:7.5	144.23	14.42	2.88	161.53	15.1	13.59	2,090.76	1,929.23	6
	3:0	0.00	0.00	3.84	3.84	2.1	1.89	290.76	286.92	12
	3:2.5	48.07	4.80	3.84	56.73	14.1	12.69	1,952.30	1,895.57	7
	3:5	96.14	9.6	3.84	109.61	12.8	11.52	1,772.30	1,662.69	8
	3:7.5	144.23	14.42	3.84	162.5	17.3	15.57	2,395.38	2,232.88	4

L = locations, IC = input cost, AC = application cost, LC = labor cost, TVC = total variable cost, MY = marketable yield, ADY = adjusted yield, GI = gross income, NB = net benefit, and USD = US dollars.

## Data Availability

The data used to support the findings of this study are included within the article.
